# Preparation of Polyoxometalate-Based Composite by Solidification of Highly Active Cobalt-Containing Polytungstate on Polymeric Ionic Liquid for the Efficient Isolation of Proteinase K

**DOI:** 10.3390/molecules28083307

**Published:** 2023-04-07

**Authors:** Jiaxuan Yang, Ning Chu, Xuwei Chen

**Affiliations:** 1 Department of Chemistry, College of Sciences, Northeastern University, Box 332, Shenyang 110819, China; y2627849213@163.com; 2 Bayuquan Customs District of the People’s Republic of China, Yingkou 115007, China; n_chu2009@sina.com

**Keywords:** polyoxometalate, water-soluble polytungstate, polymeric ionic liquid, proteinase K (PrK), isolation

## Abstract

A novel porous polyoxometalate (POM)-based composite (Co_4_PW–PDDVAC) was prepared via the solidification of water-soluble polytungstate (Co_4_PW) on the polymeric ionic liquid dimethyldodecyl-4-polyethylene benzyl ammonium chloride (PDDVAC) via a cation-exchange reaction. The solidification was confirmed by EDS, SEM, FT-IR, TGA, and so on. The strong covalent coordination and hydrogen-bonding interaction between the highly active Co^2+^ of the Co_4_PW and the aspartic acid residues of proteinase K endowed the obtained Co_4_PW–PDDVAC composite with excellent proteinase K adsorption properties. Thermodynamic investigations indicate that the adsorption behavior of proteinase K was consistent with the linear Langmuir isothermal model, giving an adsorption capacity as high as 1428 mg g^−1^. The Co_4_PW–PDDVAC composite was applied in the selective isolation of highly active proteinase K from *Tritirachium album* Limber crude enzyme fluid.

## 1. Introduction

Polyoxometalates (POMs) are a unique class of molecular inorganic compounds with unmatched electronic diversity and structural variation. Due to their strong acidity and redox properties, POMs have been widely used in the fields of electrochemistry, green chemistry, medicine, and so on [1]. Among them, POMs with the Keggin structure are the most widely studied as they exhibit the characteristics of both complexes and metallic oxides [2]. The Keggin structure consists of a central tetrahedron XO_4_ that is surrounded by a metal oxygen octahedron MO_6_, consisting of 12 shared angles. These octahedrons are divided into four M_3_O_13_ groups, each consisting of three octahedrons that share edges and a common oxygen atom [3]. Their structures are greatly affected by ambient pH conditions. There are three different lacunary anions in Keggin POMs, namely, [XM_11_O_39_]^n−^, [XM_10_O_36_]^n−^, and [XM_9_O_34_]^n−^, which have been shown to possess the advantages of high coordination activity, catalytic activity, and structural flexibility [4]. At the same time, the negative charge localization caused by the lacunary structure results in Keggin POMs with high nucleophilicity, allowing them to easily coordinate with transition-metal ions, organometallic ions, rare-earth elements, etc. [5]. Due to the “active site” and “central cation” of transition metals, such Keggin POMs generally have single-function, bifunctional, or multifunctional properties, and thus, have gained popularity in the substitution synthesis field [6].

POM Na_10_[Co_4_(H_2_O)_2_(PW_9_O_34_)_2_] (Co_4_PW) is a water-soluble “sandwich” polyoxometalate composed of a stable antioxidant polytungstate ligand Co_4_O_4_ core, and it possesses favorable hydrolytic and oxidative stability [7]. In 2010, Yin et al. demonstrated that Co_4_PW could be used as an efficient catalyst in water oxidation [8]. Cyclic voltammetry analysis showed that the catalytic current for water oxidation using Co4PW appeared near the thermodynamic potential; this could not be obtained by other cobalt-based POMs with different structures. Nagaiah et al. reported the loading of Co_4_PW with polyvinyl alcohol butylimidazole (PVIM) on N-doped carbon nanotubes and its application in metal–air batteries: in this way, Co_4_PW acted as the dual-functional catalyst for oxygen reduction and hydrogen evolution reactions because of its advantages of high conductivity and stability. The Co_4_PW-based battery exhibited a high capacity and energy density, along with an excellent recharging ability [9].

Ionic liquids (ILs) are organic salts with a melting point below 100 °C [10]. An almost unlimited number of cations and anions may combine to form ILs with an equal number of anions and cations [11]; thus, ILs appear electrically neutral at room temperature. Possessing excellent characteristics such as good thermal stability, low volatility, low toxicity, high ionic conductivity, and an adjustable electrochemical structure, ILs are known as structurally adjustable green solvents and have been widely used in various scientific research fields [12]. Polymeric ionic liquids (PILs) are polymeric chains formed by the polymerization of a repeated IL monomer. The unique properties of functionalized ILs, such as strong polarity, adjustable physicochemical properties, ionic conductivity, and thermal and chemical stability, can be well represented in PILs [13]. Thus far, PILs have been widely applied in catalysis, drug loading, electrochemistry, and energy-storage-related topics [14,15]. Owing to their high functional group density, PILs are able to produce a variety of interactions with the target object, such as van der Waals force, hydrogen bonding, electrostatic interactions, π–π stacking, etc.; this means they can be used as excellent media for adsorption separation [16], as well as in the field of sample pretreatment [17,18,19,20,21].

Proteinase K (PrK) is an important serine protein secreted by *Tritirachium album* Limber with a wide range of substrate specificities and high proteolytic activities [22], which can preferentially hydrolyze peptide bonds linked to the C terminus of sulfhydryl groups, hydrophobic amino acids, and aromatic amino acids, and can be used to degrade proteins to produce short peptides [23]. PrK consists of a peptide chain containing 277 amino acid residues with a relative molecular weight of 28930. The active sites in PrK are composed of D39, H69, and S224 with two Ca^2+^ binding sites near the active center, which contributes to the conformational stability of the protein. The substrate recognition sites are mainly G100-Y104 and S132-G136 fragments [24]. PrK exhibits high proteolytic activity towards both natural and non-natural proteins in a wide pH range, i.e., pH 4–10. Meanwhile, its proteolytic activity is well maintained even under a high level of denaturant such as urea or SDS [25]; it has, therefore, been extensively used for applications in biological detection, sewage treatment, food production, etc.

In this study, water-soluble Co_4_PW was firstly synthesized via the hydrothermal method, and polymeric ionic liquid PDDVAC was fabricated through the free-radical polymerization reaction of the ionic liquid *N*,*N*-dimethyl-dodecyl-(4-vinylbenzyl) ammonium chloride (DDVAC). A porous POM-based composite (Co_4_PW–PDDVAC) was then prepared by the solidification of water-soluble Co_4_PW on PDDVAC via the cation-exchange reaction. The solidification was confirmed using EDS, SEM, FT-IR, TGA, etc. The strong covalent coordination and hydrogen-bonding interaction between the highly active Co^2+^ of the Co_4_PW and the aspartic acid residues of PrK endowed the as-prepared Co_4_PW–PDDVAC composite with excellent PrK adsorption properties. The PrK adsorption behaviors of the Co_4_PW–PDDVAC composite, including the adsorption thermodynamics and kinetics, were investigated systematically. The Co_4_PW–PDDVAC composite exhibited a super-high theoretic adsorption capacity towards PrK, and it was used as an efficient sorbent for the selective isolation of PrK from complex matrices by using imidazole solution as the stripping reagent. An SDS-PAGE assay indicated that PrK with high activity and purity was successfully isolated from *Tritirachium album* Limber via the Co_4_PW–PDDVAC-based solid-phase extraction procedure.

## 2. Results and Discussion

### 2.1. Fabrication and Characterization of the Co_4_PW–PDDVAC Composite

**Scheme 1:**
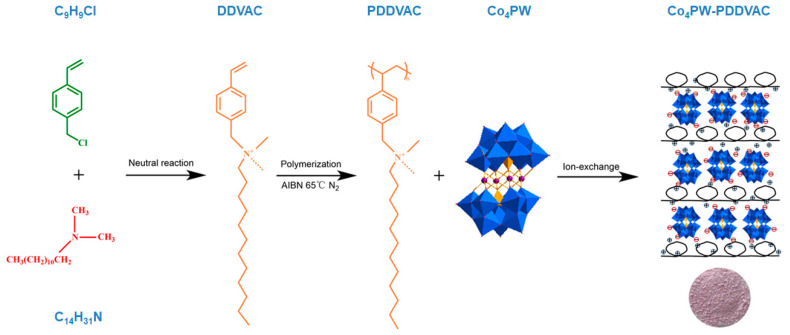
The pathway for the preparation of Co_4_PW–PDDVAC composite.

Scheme 1 illustrates the pathway for the fabrication of the Co_4_PW–PDDVAC composite. Ionic liquid monomer DDVAC was firstly obtained via the one-step neutral reaction between 4-vinyl benzyl chloride and *N*,*N*-dimethyldodecylamine, and its purity was ascertained by a 1H-NMR assay (Figure S1). The DDVAC was then polymerized into polymeric ionic liquid PDDVAC by a free-radical polymerization reaction using AIBN as the cross-linking agent, and its structure was analyzed by a 1H-NMR assay (Figure S2). Finally, the solidification of water-soluble Co_4_PW on PDDVAC was realized through the ion-exchange reaction between the PDDVAC and the polyanion [Co_4_(H_2_O)_2_(PW_9_O_34_)_2_]^10-^ Co_4_PW. The high lattice energy between the polymeric ionic liquid cation and the Co_4_PW polyanion resulted in the formation of stable precipitation. Moreover, the polyanion could combine with multiple polymeric cations via ionic bonds [26]. Therefore, a large ionic bond network was formed by multi-charge polymeric cations and polyanions. The final Co_4_PW–PDDVAC product was obtained after lyophilization and appeared as a lavender-colored powder.

**Figure 1:**
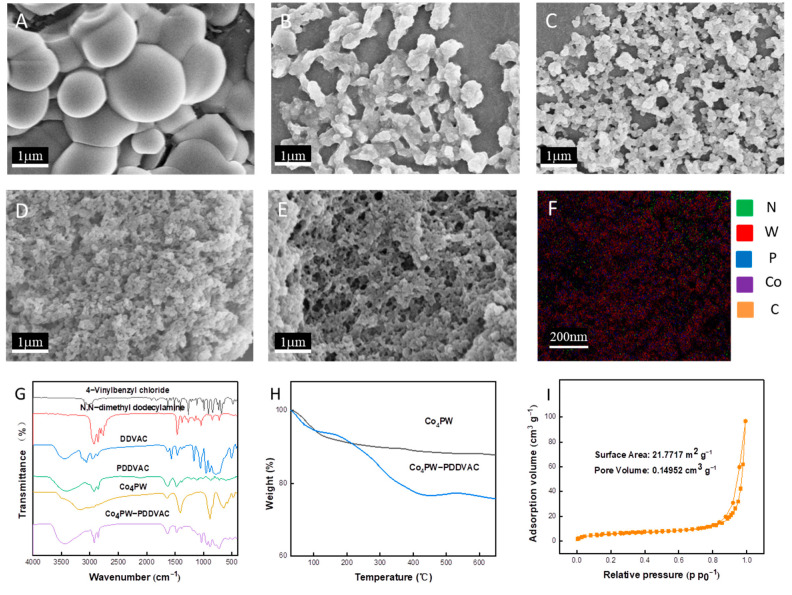
SEM images of Co_4_PW–PDDVAC composites prepared with PDDVAC/Co_4_PW molar ratio of (**A**) 1:1, (**B**) 2:1, (**C**) 5:1, (**D**) 10:1, and (**E**) 15:1. (**F**) EDS mapping of Co_4_PW–PDDVAC composite. (**G**) FTIR spectra of C_9_H_9_Cl, C_14_H_31_N, DDVAC, PDDVAC, Co_4_PW, and Co_4_PW–PDDVAC composite. (**H**) TGA curves of Co_4_PW and Co_4_PW–PDDVAC composite under N_2_ atmosphere. (**I**) N_2_ adsorption–desorption isotherms of Co_4_PW–PDDVAC composite.

Co_4_PW–PDDVAC composites were prepared under different PDDVAC/Co_4_PW molar ratios. Due to the nonrigid property of PIL and the cross-linking by ionic bonds, the Co_4_PW–PDDVAC composite prepared from a PDDVAC/Co_4_PW ratio of 1:1 was aggregated into spherical particles (Figure 1A). With the increase in the PDDVAC proportion, the size of the obtained particles decreased, and the particles linked together to form a porous structure (Figure 1B–E). This was due to the fact that the Co_4_PW content in the reaction system decreased as the proportion of PDDVAC increased, thereby reducing the cross-linking effect of the PIL molecules with a decreased POM content; thus, the suppressed aggregation of PDDVAC and the extension of the skeletons led to the formation of the network [27]. The adsorption investigation indicated that the Co_4_PW–PDDVAC composite prepared using a PDDVAC/Co_4_PW ratio of 1:5 offered the best PrK adsorption performance (Figure S3); a PDDVAC/Co_4_PW molar ratio of 1:5 was, therefore, adopted to fabricate the final Co_4_PW–PDDVAC composite for PrK adsorption.

The EDS assay indicated that the as-prepared Co_4_PW–PDDVAC composite was mainly composed of N, W, P, Co, and C elements (Figure 1F), and all elements were uniformly dispersed (Figure S4). According to the content ratio of Co to N in the obtained composites, the mass proportion of Co_4_PW in the Co_4_PW–PDDVAC composite was deduced to be 84.0% (Table S1). The FTIR spectra of C_9_H_9_Cl, C_14_H_31_N, DDVAC, PDDVAC, Co_4_PW, and Co_4_PW–PDDVAC are shown in Figure 1G. In the FTIR spectra of C_9_H_9_Cl, the stretching vibration of -C=C-H appeared at 3093 cm^−1^, and the peak of C-C on the benzene ring skeleton was located at 1409 cm^−1^. The presence of the single peak at 848 cm^−1^ indicated the para-substitution of the benzene ring. In the FTIR of C_14_H_31_N, the peaks of N-C-H and C-N appeared at 2931 cm^−1^ and 1159 cm^−1^, respectively. For DDVAC, the peaks at 3440 cm^−1^ were assigned to the C-H stretching vibration of the benzene ring. The peaks at 1169 cm^−1^ and 1045 cm^−1^ corresponded to the stretching vibration of C-N and straight chain C-C. Moreover, DDVAC showed an obvious peak at 3066 cm^−1^, which was assigned to the stretching vibration of C=C on vinyl, and which disappeared in the spectra of PDDVAC, suggesting the successful polymerization of the IL monomer. For Co_4_PW, the peaks at 1048 cm^−1^, 881 cm^−1^, 795 cm^−1^, and 948 cm^−1^ corresponded to P-O, W-O-W, and W=O, which are characteristic of the typical Keggin structure [28]. In the spectra of Co_4_PW–PDDVAC, both the characteristic peaks of PDDVAC (the vibration of benzene ring C-H at 3440 cm^−1^ and C-N at 1187 cm^−1^) and the characteristic peaks of Keggin POMs (1031 cm^−1^, 930 cm^−1^, 875cm^−1^, and 802 cm^−1^) were clearly observed, indicating that the Co_4_PW–PDDVAC composite was successfully fabricated via an ion-exchange reaction between PDDVAC and Co_4_PW.

Figure 1H illustrates the TGA curves for Co_4_PW and Co_4_PW–PDDVAC. Co_4_PW exhibited good thermal stability, and the obvious weight loss at 30–120 °C was caused by the evaporation of crystal water. As for Co_4_PW–PDDVAC, there were two weight-loss stages observed in the tested temperature range. The first stage, located within a range of 30–100 °C, corresponded to the loss of crystal water, and the second stage, located within a range of 180–430 °C, was caused by the thermal decomposition of PDDVAC. Based on the weight loss, the mass proportion of Co_4_PW on the composite was about 86.4.0%, which agreed well with that shown by EDS (84.0%).

The N_2_ adsorption–desorption isothermal curve of the Co_4_PW–PDDVAC composite (Figure 1I) conformed to the type II adsorption isotherm and was accompanied by H3 hysteresis loops [29], resulting from capillary condensation occurring within the particle. The surface area and pore volume of Co_4_PW–PDDVAC acquired via the Brunauer–Emmett–Teller (BET) determination were 21.7717 m^2^ g^−1^ and 0.14952 cm^3^ g^−1^, respectively. This indicates that the obtained composite had a large-pore structure and a wide distribution of pore size due to the generated slit pores of uneven size caused by layer stacking in the composite [30]. The large-pore structure of the Co_4_PW–PDDVAC composite offers attractive advantages for protein adsorption when compared with mesoporous [31] and microporous composites [32], as protein molecules could enter into the pore and the full surface of the Co_4_PW–PDDVAC composite is available for the retainment of target analytes.

### 2.2. The Adsorption of PrK by the Co_4_PW–PDDVAC Composite

Theoretically, the adsorption of PrK by the Co_4_PW–PDDVAC composite is based on the high-affinity coordination between Co^2+^ in polyoxometalate and the amino acid side chain of PrK. Therefore, the cobalt number in polyoxometalate will affect the protein adsorption performance of the composite material. To verify the influence of the cobalt number, three POMs with different Co numbers, CoPW ([CoH_2_OPW_11_O_39_]^5−^), Co_4_PW, and Co_9_PW ([Co_9_(OH)_3_(H_2_O)_6_(HPO_4_)_2_(PW_9_O_34_)_3_]^16−^), were prepared and solidified on PDDVAC, and the adsorption performance of the obtained CoPW–PDDVAC, Co_4_PW–PDDVAC, and Co_9_PW–PDDVAC composites was studied.

**Figure 2:**
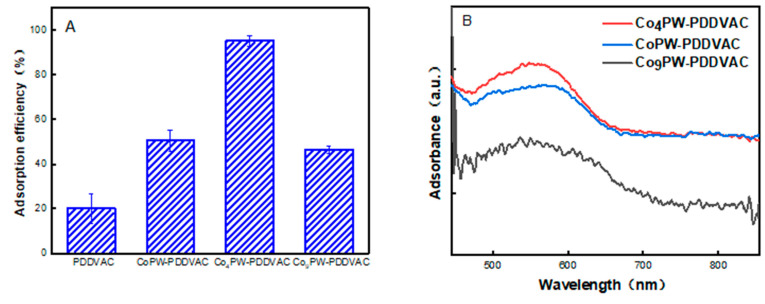
(**A**) The PrK adsorption performance of PDDVAC, CoPW–PDDVAC, Co_4_PW–PDDVAC, and Co_9_PW–PDDVAC. (**B**) Ultraviolet diffuse spectra of CoPW–PDDVAC, Co_4_PW–PDDVAC, and Co_9_PW–PDDVAC.

The PrK adsorption efficiency of these three composites is shown in Figure 2A. It can be seen that the adsorption of PrK by the polymeric ionic liquid PDDVAC was poor. The three POM-based composites all showed improved adsorption performance, indicating that the polyoxometalate components in the composites contributed greatly to the adsorption of PrK. Interestingly, Co_4_PW–PDDVAC exhibited the best adsorption of PrK among the three composites. Table S1 shows the element composition of these three composites, which was acquired by EDS analysis. It can be seen that the Co contents of CoPW–PDDVAC, Co_4_PW–PDDVAC, and Co_9_PW–PDDVAC were 4.00%, 5.00%, and 6.00%, respectively. This suggests that the Co content in the composites is closely related to the Co^2+^ number in the polyoxometalate. However, it is worth noting that Co_9_PW–PDDVAC, with the highest Co content, exhibited a lower adsorption efficiency than Co_4_PW–PDDVAC; thus, it is speculated that the Co^2+^ in the three composites might exist in different coordination states.

Ultraviolet diffuse spectra can be used to study the structure, oxidation state, and coordination state of transition-metal ions and their complexes. Therefore, in order to confirm this speculation, the ultraviolet diffuse spectra of these three composites were recorded (Figure 2B). It can be seen that wide reflection bands centered at 550 cm^−1^ were found for all composites, which is related to the Co^2+^ sites from the Co-O-W entities of the polyoxometalate [33]. Among these three composites, Co_4_PW–PDDVAC exhibited the reflection band with the highest intensity, suggesting that Co^2+^ in Co_4_PW–PDDVAC is the most active and easily coordinates with target analytes. Although the Co_9_PW–PDDVAC composite possessed the highest Co content, it offered the lowest reflection intensity, which might be attributed to the steric hindrance of (PW_9_O_34_)^9−^ groups [34]. It is obvious that the activity of Co^2+^ in the three composites followed the sequence of Co_4_PW–PDDVAC > CoPW–PDDVAC > Co_9_PW–PDDVAC, which is consistent with the protein absorption efficiency order.

**Figure 3:**
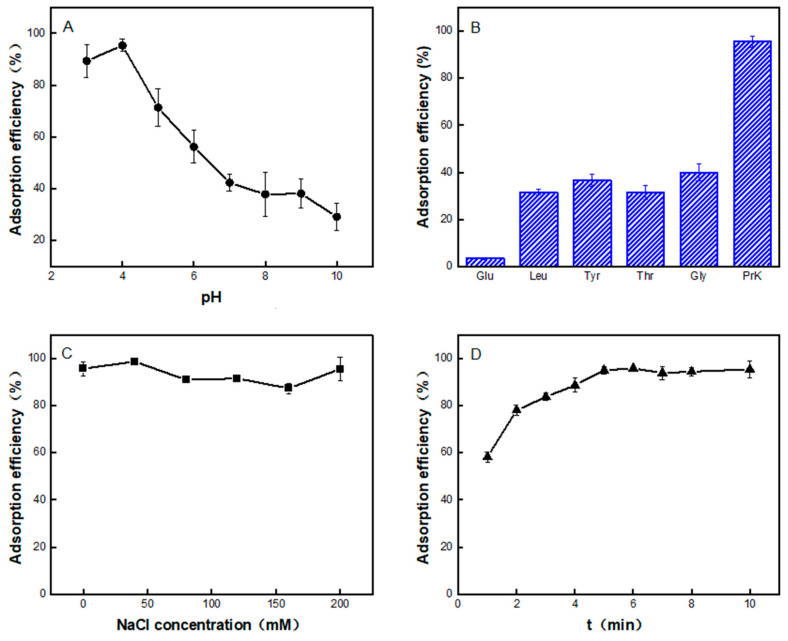
(**A**) Effect of pH on PrK adsorption efficiency. (**B**) Adsorption efficiency of Glu, Leu, Tyr, Thr, Gly, and PrK on Co_4_PW–PDDVAC composite. Effect of ionic (**C**) strength and (**D**) adsorption time on PrK adsorption efficiency. Protein solution: 100 μg mL^−1^, 1.0 mL; Co_4_PW–PDDVAC composite: 0.2 mg; pH: 4.0.

**Figure 4:**
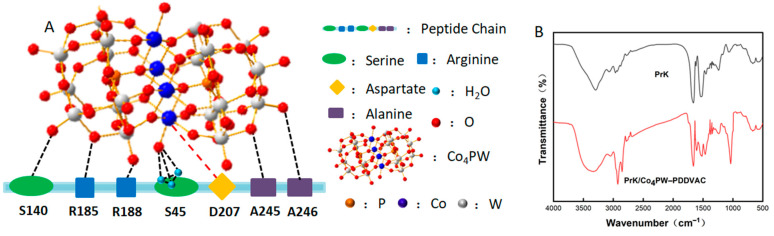
(**A**) Schematic illustration of the interactions between Co_4_PW–PDDVAC composite and PrK peptide chains. (**B**) FTIR spectra of PrK and PrK/Co_4_PW–PDDVAC.

The effect of pH value on the adsorption of PrK by the Co_4_PW–PDDVAC composite was investigated in a pH range of 3–10; the results indicate that the best adsorption of PrK was achieved under a pH of 4.0 (Figure 3A). These PrK adsorption behaviors might be explained by the coordinating interaction and the formation of multiple hydrogen bonds between PrK and the Co_4_PW–PDDVAC composite, as illustrated in Figure 4A. PrK is a well-defined spherical α/β protein species containing 6 α-helices, 15 β-folds, and a 3/10 helix [35]. The aspartic acid residues at D207 of the PrK peptide chain can covalently coordinate with the Co^2+^ in Co_4_PW under acidic conditions. At the same time, there is a strong hydrogen-bonding interaction between the hydroxyl-H on the side chain of amino acid residues near D207, such as S140, R185, R188, A245, and A246, and the terminal-O of [PW_9_O_34_]^9−^ in the Keggin structure [36]. Moreover, there is a hydrogen bond network between the terminal oxo-ligand directly connected to Co^2+^ in Co_4_PW and the hydrating water molecules on the surface of the S45 serine on the PrK chain. These oxo-ligands can act as both H-acceptors and H-donors, and thus they are more flexible in forming hydrogen bonds than the terminal oxo-ligands in unsubstituted Keggin structures [37].

FTIR spectra can provide information about hydrogen bonds [38,39] and the effect of the interaction between metal ions and ligand groups. In order to verify the interaction between the Co_4_PW–PDDVAC composite and proteinase K, The FTIR spectra of PrK in the absence/presence of Co_4_PW–PDDVAC were recorded (Figure 4B). The stretching vibrations of -NH at 3301 cm^−1^ and 3072 cm^−1^ for PrK shifted to 3309 cm^−1^ and 3060 cm^−1^ after the addition of Co_4_PW–PDDVAC due to the formation of the coordination bond between the nitrogen atom and cobalt ions [40]. The vibration peak attributed to the C = O stretching at 1668 cm^−1^ shifted to 1666 cm^−1^, and the peak of -COOH shifted from 1444 cm^−1^ to 1469 cm^−1^ due to the replacement of hydrogen by cobalt. In addition, the peak attributed to the stretching vibration of C-O changed from 1078 cm^−1^ to 1045 cm^−1^. The results of the FTIR spectra suggested that the interaction between the Co_4_PW–PDDVAC composite and proteinase K was mainly due to the coordination of C_O_^2+^ in the Co_4_PW–PDDVAC composite with the carboxyl, carbonyl, and amino groups in the D207 aspartic acid of PrK.

Under acidic conditions, the oxo-ligand connected to Co^2+^ in the Keggin structure of a composite is easy to protonate, which promotes its coordination with the carboxylic acid side chain of amino acid residue D207 on PrK. Therefore, the adsorption efficiency of PrK gradually increases with the decrease in pH value. However, the high protonation of the oxo-ligand connected to the Co^2+^ and terminal oxygen in the [PW_9_O_34_]^9−^ structure under strong acidic conditions was not favorable for the formation of hydrogen bonds [41]; accordingly, the adsorption efficiency decreases when the pH was lower than 4.0. Therefore, a BR buffer of a pH of 4.0 was adopted for the sample preparation.

Figure 3B shows the adsorption performance of the Co_4_PW–PDDVAC composite towards other protein/acid amino species, including glucose (Glu), leucine (Leu), tyrosine (Tyr), threonine (Thr), and glycine (Gly). It is clear that the adsorption of PrK was much better than that of other species, suggesting the favorable adsorption selectivity of the Co_4_PW–PDDVAC composite towards PrK.

As there are various electrolytes existing in real biological samples, the influence of ionic strength on PrK adsorption was studied by adding different concentrations of NaCl into the protein samples. As shown in Figure 3C, the variation in NaCl concentration in the range of 0–200 mmol L^−1^ barely influenced PrK adsorption, demonstrating that the electrostatic interaction made a negligible contribution to PrK adsorption, and PrK adsorption would not be affected by electrolytes in real samples.

Figure 3D shows the effect of adsorption time on PrK adsorption. Adsorption efficiency first increased with time and then stabilized once the adsorption time surpassed 5 min. For a comprehensive consideration of adsorption efficiency and time cost, an adsorption time of 5 min was thus adopted. Compared with the conventional gel chromatographic column-based separation process, the adsorption time was reduced greatly due to the high-affinity interaction.

### 2.3. Thermodynamic Analysis of PrK Adsorption by the Co_4_PW–PDDVAC Composite

The thermodynamic properties of the PrK adsorption process were investigated through thermodynamic parameters such as enthalpy change (∆*H*^0^), entropy change (∆*S*^0^), and Gibbs free energy (∆*G*^0^), which were calculated using the following formulas [42,43]:

(1) $$\Delta G^{0}=\Delta H-T\Delta S^{0}$$

(2) $$Lnk_{d}^{}=\frac{\Delta S^{0}}{R}-\frac{\Delta H^{0}}{RT}$$

where *R* is the molar gas constant (8.314 J mol^−1^ K^−1^); T is the temperature (K); ∆*G*^0^ is the Gibbs free energy (kJ mol^−1^); ∆*H*^0^ is the adsorption enthalpy (kJ mol^−1^); ∆*S*^0^ is the adsorption entropy variable (J mol^−1^); and *k_d_* stands for the thermodynamic adsorption equilibrium constant.

In order to explore the adsorption process of PrK from the perspective of thermodynamics, the amount of adsorbed PrK on the Co_4_PW–PDDVAC composite was measured under temperatures of 288, 293, 303, 318, and 323 K. As shown in Figure S5A, the amount of adsorbed PrK decreased with temperature. The functions of the distribution coefficient with temperature are demonstrated in Figure S5B, and Table 1 lists the detailed thermodynamic parameters. The positive Δ*H*^0^ indicates that the PrK adsorption process was radiative. Δ*G*^0^ was negative in the tested temperature range, revealing that the adsorption process was spontaneous and possessed good thermodynamic properties. The increase in Δ*G*^0^ with temperature suggests that the adsorption of PrK decreased with temperature. The negative value of Δ*S*^0^ indicates the decreased disorder and randomness at the solid–solution interface [44].

**Figure 5:**
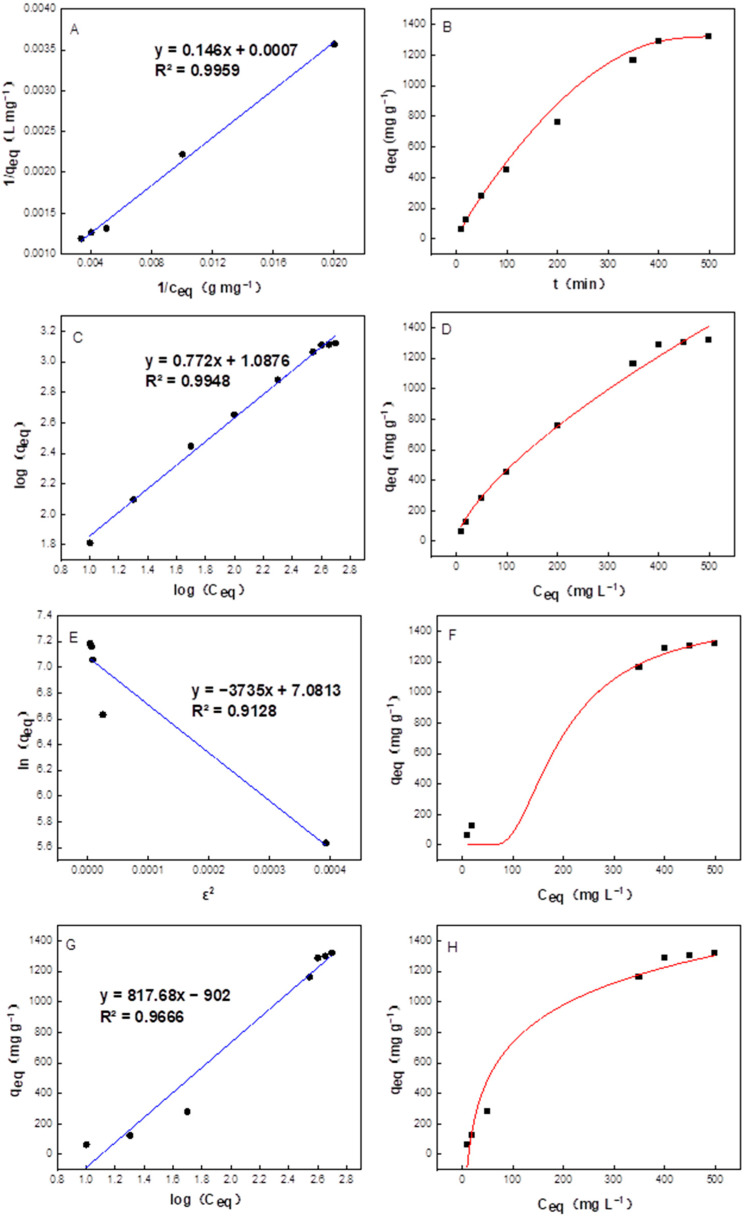
Linear and nonlinear plots of Langmuir isotherm model (**A**,**B**), Freundlich isotherm model (**C**,**D**), Dubinin–Radushkevich isotherm model (**E**,**F**), and Temkin isotherm model (**G**,**H**) of PrK adsorption by Co_4_PW–PDDVAC composite.

To evaluate the PrK adsorption capacity of the Co_4_PW–PDDVAC composite, the adsorption behavior of the Co_4_PW–PDDVAC composite with different concentrations of PrK (50–400 mg L^−1^) was studied. Langmuir, Freundlich, Dubinin–Radushkevich, and Temkin adsorption isothermal models were used to fit the experimental data linearly and nonlinearly. The fitting results are shown in Figure 5, and the relevant model parameters are summarized in Table 2.

The Langmuir model assumes that the surface properties of adsorbents are homogeneous in nature and monolayer adsorption prevails at each site [45]. The significance of the Langmuir model is not only in its simple mathematical form and its ability to represent the first type of adsorption isotherm, but also in the fact that it comprises the multimolecular layer adsorption theory proposed later. The linear and nonlinear equations of the Langmuir model can be expressed as

(3) $$q_{eq}=\frac{Q_{m}b_{L}C_{eq}}{1+b_{L}C_{eq}}$$

(4) $$\frac{1}{q_{eq}}=\frac{1}{Q_{m}b_{L}}\left(\frac{1}{C_{eq}}\right)+\frac{1}{Q_{m}}$$

where *Q_m_* is the maximum adsorption capacity (mg g^−1^) and *b_L_* is the Langmuir constant (L mg^−1^).

Figure 5A,B shows the linear and nonlinear fitting results of the Langmuir model. Both fitting models presented high correlation coefficients, with the correlation coefficient of linear fitting (R^2^ = 0.9959) being slightly higher than that of nonlinear fitting (R^2^ = 0.9951); thus, it is presumed that the PrK adsorption behavior of Co_4_PW–PDDVAC was in good correlation with the Langmuir isotherm.

The Freundlich model is an adsorption equilibrium formula based on the adsorption of adsorbents on heterogeneous surfaces. This model considers that the binding site on the surface of adsorbents is highly heterogeneous and the effect of adsorption heat is ignored [46]. This model is appropriate for the analysis of multilayer adsorption and surface adsorption under nonideal conditions. The Freundlich isotherm in the form of nonlinear and linear formulas is given in Equations (5) and (6), respectively:

(5) $$q_{eq}=K_{F}C_{eq}{}^{1/n}$$

(6) $$\log q_{eq}=\log K_{F}+\frac{1}{n}\log C_{eq}$$

where *K_F_* is the Freundlich constant (mg g^−1^); 1/*n* is the adsorption strength, which varies with the material heterogeneity (L g^−1^); and n is a constant, which is usually greater than 1. The larger the value of *n*, the more obvious the nonlinearity of the adsorption isotherm. *n* < 10 signifies a favorable adsorption. Conversely, *n* > 10 means that the adsorption isotherm has become a rectangular isotherm or an irreversible isotherm due to the requirement that the concentration of the adsorbed molecule must be reduced sufficiently when it is desorbed from the surface.

As shown in Figure 5C,D, the linear fitting correlation coefficient of the Freundlich model (R^2^ = 0.9948) was higher than the nonlinear correlation coefficient (R^2^ = 0.9899); this illustrates that the linear model conformed to the adsorption process better than the nonlinear model.

The D-R isotherm model was established based on the microporous filling theory and is suitable for the adsorption process of microporous materials. Its premise is that the adsorption process does not consist of layered adsorption but rather pore-filled adsorption, and is controlled by the adsorption field in the micropore space. The D-R isotherm model, in its nonlinear and linear forms, is denoted by the following equations [47]:

(7) $$q_{eq}=Q_{\max}\exp(-\beta \varepsilon^{2})$$

(8) $$\ln q_{eq}=\ln Q_{\max}-\beta \varepsilon^{2}$$

where *β* is the adsorption energy constant (mol ^2^ KJ^−2^) and ε is the adsorption potential, expressed as RT ln(1 + 1/*C_eq_*). The correlation coefficients calculated by this model were relatively low (Figure 5E,F), suggesting that the PrK adsorption process by Co_4_PW–PDDVAC poorly matched the D-R model.

The Temkin model assumes that there is an interaction force within the adsorbent in the adsorption process [48]. Equations (9) and (10) represent the linear and nonlinear expressions of the Temkin isotherm:

(9) $$q_{eq}=\frac{RT}{B_{T}}\ln(K_{T}C_{eq})$$

(10) $$q_{eq}=\frac{RT}{B_{T}}\log C_{eq}+\frac{RT}{B_{T}}\log K_{{}_{T}}$$

where *R* is the universal gas constant (KJ mol^−1^ K^−1^); T is the temperature (K); *K_T_* is the binding constant for the maximum binding energy (L mg^−1^); and *B_T_* is the adsorption heat (KJ mol^−1^).

Figure 5G,H shows the linear and nonlinear fitting results of the Temkin model: their correlation coefficients were low, and it is presumed that the PrK adsorption process by the Co_4_PW–PDDVAC composite did not conform to the Temkin model.

Therefore, the selection of the best isotherm model for a Co_4_PW–PDDVAC-based adsorption system can be interpreted as follows: linear Langmuir (R^2^ = 0.9959) > nonlinear Langmuir (R^2^ = 0.9951) > linear Freundlich (R^2^ = 0.9948) > nonlinear Freundlich (R^2^ = 0.9899). It can be concluded that the PrK adsorption of the Co_4_PW–PDDVAC composite conformed to monolayer adsorption. The theoretical PrK adsorption capacity of Co_4_PW–PDDVAC was derived as 1428.0 mg g^−1^.

### 2.4. Kinetic Analysis of PrK Adsorption by the Co_4_PW–PDDVAC Composite

Adsorption kinetics can illustrate the adsorption rate of the adsorbent to adsorbate; this determines the retention time of the adsorbate at the solid–liquid interface. The adsorption time directly affects the efficiency of the adsorbent. Therefore, adsorption kinetics data are an important reference basis in the design of the adsorption process. To further probe the adsorption operation, four kinetic models including the pseudo-first-order, pseudo-second-order, intraparticle, and Elovich models in both their linear and nonlinear forms were employed to fit the experimental data.

The pseudo-first-order kinetic model [49] was based on the concept of solid adsorption capacity; the adsorption process is described from the perspective of diffusion using the following formulas:

(11) $$q_{t}=q_{eq}\left(1-e^{-k_{1}t}\right)$$

(12) $$\log(q_{eq}-q_{t})=\log q_{eq}-((K_{1}/2.303)t)$$

where *q_eq_* is the adsorption capacity of the adsorbent at equilibrium (mg g^−1^); *q_t_* is the adsorption capacity of the adsorbent at a different time (mg g^−1^); *k_1_* is the rate constant (h^−1^) of the pseudo-first-order dynamic model; and *t* is the adsorption time (h).

The pseudo-second-order dynamic model was proposed to expound on chemisorption-based adsorption processes. The formula is described in the following equation:

(13) $$\frac{t}{q_{t}}=\frac{1}{k_{2}q_{e}^{2}}+\frac{t}{q_{e}}$$

where *k*_2_ is the rate constant (h^−1^) of the pseudo-second-order dynamic model (g mg^−1^ min^−1^).

The intraparticle kinetics model hypothesizes that the adsorption process is split into three steps: the liquid-film diffusion stage, intragranular diffusion stage, and internal surface adsorption stage. The intraparticle kinetic model equation is expressed by Equation (14):

(14) $$q_{t}=K_{3}t^{1/2}+C$$

where *K*_3_ is the diffusion rate constant (mg g^−1^ min^1/2^), and the thickness of the boundary layer is signified by *C*. The values of *K*_3_ and *C* can be calculated by plotting a linear plot of *q_t_* versus *t*^1/2^.

The Elovich kinetic model is used to describe the chemisorption process, which assumes that the adsorption site is multiphase and shows different activation energies in the adsorption process [50]. Based on the empirical formula, this model can be simplified into the linear relationship between qt and ln*t*, as shown in the formula:

(15) $$q_{t}=(1/\beta )\ln(\alpha \beta )+(1/\beta )\ln t$$

where *α* is the constant related to the chemisorption rate (mg min g^−1^) and *β* is the constant related to the surface coverage degree (g mg^−1^).

Figure S6 illustrates the obtained adsorption kinetic curves; the relevant fitting parameters are summarized in Table 3. The correlation coefficients of the intraparticle and Elovich models were relatively low, suggesting that the PrK adsorption behaviors of the Co_4_PW–PDDVAC composite did not match these models.

Hence, based on the comparisons between the linear and nonlinear forms of the pseudo-first-order and pseudo-second-order models, it was concluded that the order of best fit for the Co_4_PW–PDDVAC adsorption system was as follows:

Linear pseudo-second-order (R^2^ = 0.9980) > linear pseudo-first-order (R^2^ = 0.9944) > nonlinear pseudo-second-order (R^2^ = 0.9720) > nonlinear pseudo-first-order (R^2^ = 0.9370).

The results clearly indicate that the PrK adsorption process by the Co_4_PW–PDDVAC composite was affected by chemisorption and liquid-film diffusion, with chemisorption dominating the PrK adsorption process. This was consistent with the above-mentioned adsorption mechanism where the coordination and hydrogen-binding interaction were deduced to be the main driving forces behind PrK adsorption.

### 2.5. Recovery of Adsorbed PrK from the Co_4_PW–PDDVAC Composite

**Figure 6:**
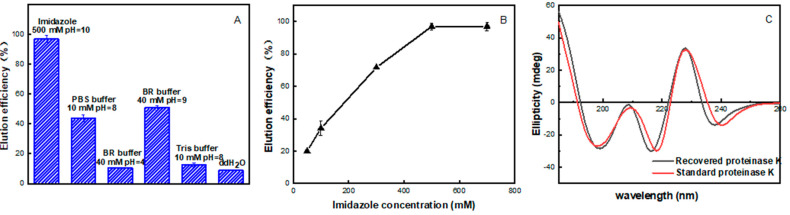
(**A**) Recovery of the adsorbed PrK using various stripping reagents. (**B**) Effect of imidazole concentration on elution efficiency. (**C**) Circular dichroic spectra of PrK. Protein solution: 100 μg mL^−1^, 1.0 mL; pH 10.0; Co_4_PW–PDDVAC composite: 0.2 mg; stripping time: 30 min; stripping reagent: 500 mmol L^−1^, 1.0 mL.

In order to recover the retained PrK from the surface of the Co_4_PW–PDDVAC composite, the eluting performance of several reagents including an imidazole solution (500 mM, pH = 10), PBS (10 mM, pH = 8), BR (40 mM, pH = 4), BR (40 mM, pH = 9), Tris (10 mM, pH = 8), and deionized water was investigated. As shown in Figure 6A, the highest elution efficiency was obtained using the imidazole solution as the stripping reagent. This might be because imidazole can undergo coordination complexation with Co^2+^ and compete with the Co_4_PW–PDDVAC composite to achieve elution of the adsorbed PrK. Figure 6B shows the effect of imidazole concentration on elution efficiency. The elution efficiency obviously increased as the imidazole concentration changed from 50 mmol L^−1^ to 500 mmol L^−1^; therefore, 500 mmol L^−1^ of imidazole was selected for this study.

In order to assess the effect of the adsorption–elution process on the structure of PrK, circular dichroic spectra (CD) analysis was performed. As shown in Figure 6C, standard PrK exhibited a positive peak at 189 nm and an obvious negative peak at 200 nm, which was attributed to the α-helix structure of the protein. The CD spectrum of recovered PrK coincided with that of standard PrK, suggesting that the Co_4_PW–PDDVAC-based isolation process did not affect the secondary structure of the protein.

### 2.6. Reusability and Stability of the Co_4_PW–PDDVAC Composite

**Figure 7:**
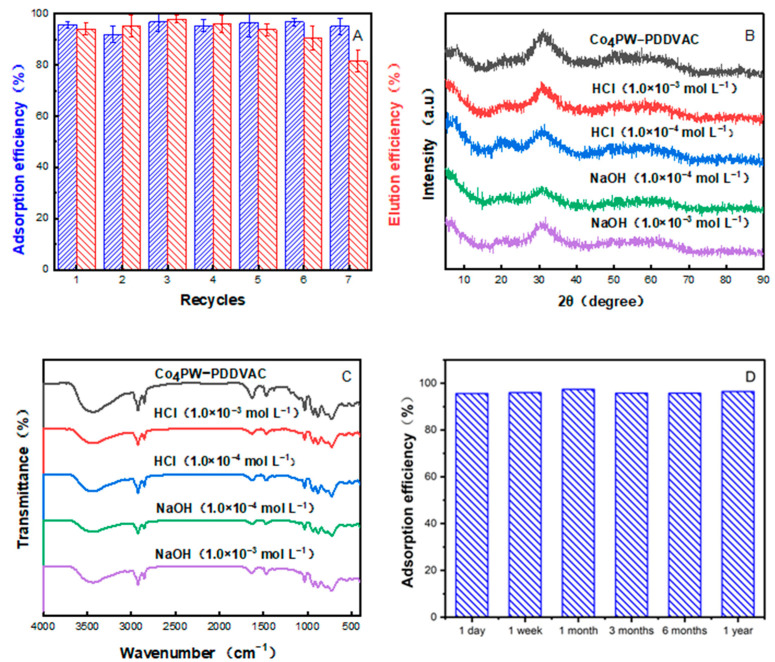
(**A**) The reusability of Co_4_PW–PDDVAC composite. XRD patterns (**B**) and FTIR spectra (**C**) of Co_4_PW–PDDVAC composites under different chemical circumstances for 24 h. (**D**) PrK adsorption efficiency by Co_4_PW–PDDVAC composite at different storage times.

Reusability is an important index to evaluate the practical potential of sorbent materials. The PrK adsorption performance of the Co_4_PW–PDDVAC composite for continuous adsorption–desorption processes is shown in Figure 7A. During the five adsorbing–eluting cycles, no obvious change was observed for the adsorption and elution efficiency of PrK, suggesting that the Co_4_PW–PDDVAC composite possessed favorable reusability.

The chemical stability of the Co_4_PW–PDDVAC composite was investigated by placing the sorbent in different acidic/alkali environments and leaving it to stand for 24 h. Figure 7B,C illustrates the recorded XRD patterns and FT-IR spectra. Compared to the pristine composite, hardly any changes were observed in the XRD and FTIR spectra of the composite after treatment with an acid and base, revealing the good chemical stability of the Co_4_PW–PDDVAC composite.

The PrK adsorption performance by the Co_4_PW–PDDVAC composite after storage at different times is shown in Figure 7D. The PrK adsorption efficiency remained above 90% even after 12 months of storage, suggesting that the Co_4_PW–PDDVAC composite possessed excellent long-term stability.

### 2.7. Isolation of PrK from Tritirachium album Limber

**Figure 8:**
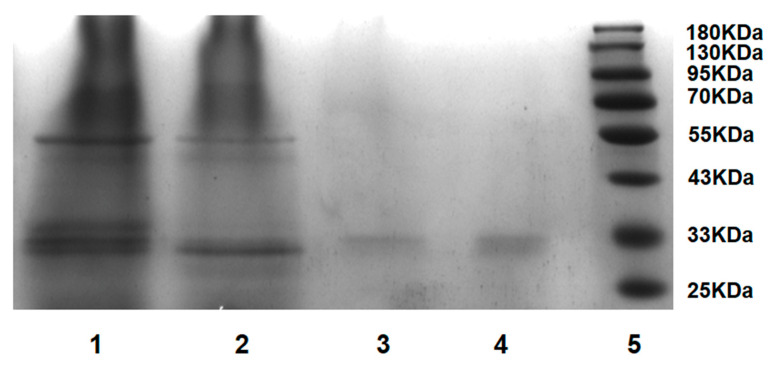
SDS-PAGE analysis of purified PrK. Lane 1: *Tritirachium album* Limber crude enzyme fluid; Lane 2: supernatant after adsorption by Co_4_PW–PDDVAC composite; Lane 3: recovered solution; Lane 4: standard PrK (500 μg mL^−1^); Lane 5: protein marker.

In order to evaluate the practical application of the Co_4_PW–PDDVAC composite, it was used to isolate PrK from *Tritirachium album* Limber. An amount of 1 mL of *Tritirachium album* Limber crude enzyme fluid was treated with the optimized adsorption–elution process. Figure 8 illustrates the SDS-PAGE assay results. Lane 1 was the crude enzyme fluid of *Tritirachium album* Limber: it had several clear bands in the range of 25–70 KDa due to the coexistence of various protein species. Lane 2 was the supernatant after adsorption by the Co_4_PW–PDDVAC composite. Only one protein band appeared in the recovered solution (Lane 3), which was located at the same position as that of standard PrK (Lane 4). This suggests that PrK of high purity was successfully isolated from the crude enzyme fluid using the Co_4_PW–PDDVAC-based solid-phase extraction procedure.

Table 4 compares the performance of the commercial procedure and the Co_4_PW–PDDVAC-based solid-phase extraction procedure for PrK isolation. It can be seen that the Co_4_PW–PDDVAC-based solid-phase extraction has the advantages of a reduced time and high protein yield, and these might provide a feasible strategy for the large-scale production of highly purified PrK.

## 3. Materials and Methods

### 3.1. Chemicals and Reagents

Proteinase K (PrK, pI 8.9, Mr 28.9 kDa, P2308, ≥99%), glycine (C_2_H_5_NO_2_, ≥99%), *N*,*N*,*N*,*N*-tetramethylethylenediamine (TEMED, ≥99%), ammonium persulphate (APS, 98%), tris (hydroxymethyl) aminomethane (Tris, >99.9%), acrylamide *N*,*N*′-methylene-bis-acrylamide, and sodium dodecyl sulphate (SDS, ≥99%) were purchased from Sigma-Aldrich (St. Louis, MO, USA) and used without further purification.

Other reagents including Coomassie Brilliant Blue (G-250, ≥99%), 4-vinyl benzyl chloride (C_9_H_9_Cl, ≥90%), *N*,*N*-dimethyldodecylamine (C_14_H_31_N, ≥96%), sodium tungstate dihydrate (Na_2_WO_4_⋅2H_2_O, ≥99.9%), 2,2′-Azobis(2-methylpropionitrile) (C_8_H_12_N_4_, ≥99%), cobalt acetate tetrahydrate (Co(OAC)_2_·4H_2_O, ≥99.9%), sodium hydroxide (NaOH, ≥99%), hydrochloric acid (HCl, ≥99%), phosphoric acid (H_3_PO_4_, ≥99%), boric acid (H_3_BO_3_, ≥99%), acetic acid (HAc, ≥99%), imidazole, sodium chloride (NaCl, ≥99%), ethyl acetate (C_4_H_8_O_2_, ≥99.5%), potassium phosphate monobasic (KH_2_PO_4_, ≥99%), sodium phosphate dibasic (Na_2_HPO_4_, ≥99%), and ethanol (C_2_H_5_OH, ≥99%) were provided by Aladdin (Shanghai, China). Deionized water (ddH_2_O) of 18 MΩ cm was used throughout. The fungus *Tritirachium album* Limber was provided by the CBS Filamentous Fungi and Yeast Collection (CBS 368.72).

### 3.2. Synthesis of Water-Soluble Polyoxometalate Co_4_PW

Quantities of 5.09 g (15.4 mmol) of sodium tungstate dihydrate, 0.307 g (1.71 mmol) of sodium phosphate dibasic, and 0.851 g (3.42 mmol) of cobalt acetate tetrahydrate were mixed and dissolved in 35 mL of deionized water. The pH of the solution was then adjusted to 7.0 with concentrated hydrochloric acid to obtain a purple suspension. Thereafter, the suspension was heated to 100 °C, reflowed for 2 h, and filtered with a 0.25 μm filter membrane. The obtained solution was saturated with NaCl, cooled to room temperature, and crystallized for 4 h. Finally, the purple crystal Co_4_PW was collected, quickly washed 3 times with deionized water, and then dried under vacuum at 50 °C.

### 3.3. Preparation of Polymeric Ionic Liquid PPDVAC

A quantity of 15.3 g (100 mmol) of 4-vinylbenzyl chloride was dissolved in 50 mL of ethanol, and the solution was maintained under stirring in an ice bath. Next, 21.3g (100 mmol) of *N*,*N*-dimethyldodecylamine dissolved in 50 mL of ethanol was slowly added. The resultant mixture was stirred at 0 °C for 2 h and further stirred at 25 °C for 24 h. After the reaction, the solvent was removed via rotary evaporation, and the produced yellowish-green viscous liquid was recrystallized with ethyl acetate and dried under vacuum at room temperature to produce the ionic liquid *N*,*N*-dimethyl-dodecyl-(4-vinylbenzyl) ammonium chloride (DDVAC).

Following this, 4.5 g (15 mmol) of DDVAC and 24.6 mg (0.15 mmol) of AIBN were dissolved in 15 mL of ethanol. The polymerization reaction was performed at 65 °C for 24 h under a nitrogen atmosphere, and the system was then cooled to room temperature. The produced polymer was precipitated in deionized water, filtered with a 0.25 μm filter membrane, and dried under vacuum at 50 °C to obtain the polymeric ionic liquid PDDVAC product.

### 3.4. Fabrication of the Co_4_PW–PDDVAC Composite

An amount of 200 mg of PDDVAC was dissolved in 20 mL of ethanol and mixed with 20 mL of Co_4_PW solution (30 mg mL^−1^). The obtained mixture was heated to 60 °C, refluxed for 2 h, then transferred to a PTFE reactor and heated at 90 °C for 24 h. Thereafter, the produced precipitate was collected via centrifugation, washed with deionized water and ethanol, and freeze-dried under vacuum at −50 °C, giving the final lavender powder Co_4_PW–PDDVAC composite product. The obtained Co_4_PW–PDDVAC composite was placed in a glass vial, sealed with Parafilm, and stored at room temperature.

### 3.5. Characterization of the Co_4_PW–PDDVAC Composite

FT-IR spectra were recorded on a Nicolet 6700 spectrometer (Thermo Electron, Waltham, MA, USA) using a KBr disk from 400 to 4000 cm^−1^ with a resolution of 2.0 cm^−1^. X-ray diffraction (XRD) patterns were obtained on a Rigaku D/max-a X-ray diffractometer (Rigaku, Tokyo, Japan) with graphite-monochromatized Cu-Kα radiation (k = 1.54056 Å) and with a step size of 0.03°. Thermogravimetric analysis (TGA) was performed on a TGA/DSC 1/1600 LF Thermogravimetric Analyzer (Mettler Tolede, Greifensee, Switzerland) with a temperature range from 25 to 800 °C. Scanning electron microscopic (SEM) images were obtained on a SU8010 field-emission electron microscope with S-3 and a voltage of 5.0 KV (Hitachi, Tokyo, Japan). A U-3900 UV–vis spectrophotometer (Hitachi, Japan) with a 10 mm quartz cell was used for the quantitative detection of proteinase K. Circular dichroism (CD) spectra in the wavelength range of 180–260 nm were obtained on an MOS-450 automatic recording spectropolarimeter (Bio-Logic, Seyssinet-Pariset, France) with nitrogen protection at a scan rate of 0.5 s nm^−1^.

### 3.6. PrK Adsorption by the Co_4_PW–PDDVAC Composite

A quantity of 0.2 mg of the Co_4_PW–PDDVAC composite was mixed with 1.0 mL of the PrK solution (prepared in 0.04 mol L^−1^ of BR buffer, pH 4.0) and shaken for 5 min to facilitate protein adsorption. After centrifugation at 8000 rpm for 5 min, the supernatant was collected and the residual protein content in the supernatant was determined via the Bradford method by monitoring the absorbance change at 595 nm. According to the protein concentration before and after adsorption, the following equation was used to calculate the adsorption efficiency E_1_ of PrK:

(16) $$E_{1}=\frac{C_{0}-C_{1}}{C_{0}}\times 100\%$$

where *C_0_* and *C*_1_ represent the original and the residual protein concentrations, respectively.

The Co_4_PW–PDDVAC composite after adsorption was prewashed with 1.0 mL of deionized water to remove any loosely retained species on the surface of the adsorbent. Subsequently, 1.0 mL of imidazole solution (0.5 mol L^−1^, pH 10.0) was added and the mixture was oscillated for 30 min to strip the adsorbed protein from the surface of the Co_4_PW–PDDVAC composite. The supernatant, after centrifugation at 8000 rpm for 5 min, was collected to evaluate the elution efficiency and for use in the ensuing investigations.

### 3.7. Determination of PrK Activity

The activity of PrK was measured by the Kunitz method [52]. The principle is that PrK can hydrolyze casein to produce L-tyrosine, which exhibits ultraviolet absorption at 275 nm. The specific procedure was as follows: 100 μL of PrK solution was preheated in a water bath at 55 °C for 2 min, then mixed with 100 μL of casein solution (10 mg L^−1^). For the reaction, the mixture was maintained for 5 min at 55 °C in the water bath and 200μL of trichloroacetic acid solution (0.4 mol L^−1^) was then added to terminate the reaction. After standing for 5 min, the system was filtered with a 0.25 μm filter membrane and the absorbance of the collected filtrate at 275 nm was recorded.

### 3.8. Isolation of PrK from Tritirachium album Limber

The medium for propagation culturing was prepared as follows: 3.5 g of PDB medium powder was dissolved in 100 mL of deionized water, boiled, and sterilized at 121 °C in an autoclave for 20 min, and cooled to room temperature in a biosafety cabinet. After inoculation of the fungi in the medium, the system was oscillated on an oscillator (26 °C, 250 rpm) for about 120 h, and then left at 4 °C for further use.

The medium for proteinase production was prepared according to a previously reported method [51]. Briefly, 1.0 g of casein peptone, 0.01 g of MgSO_4_⋅7H_2_O, 0.005 g of CaCl_2_⋅2H_2_O, 0.28 g of KH_2_PO_4_, and 0.07 g of Na_2_HPO_4_⋅12H_2_O were dissolved in 100 mL of deionized water. Sterilization was carried out in an autoclave at 121 °C for 20 min. Following this, 1.0 g of glucose was added to the cooled system. After inoculation of the fungi in the medium for proteinase production, the system was shaken on an oscillator (26 °C, 200 rpm) for about 96 h, and then left at 4 °C.

For the isolation of PrK, the bacterial solution in the proteinase production medium was centrifugated at 4 °C under 2800 rpm for 20 min. After filtration using a 0.45 µm aseptic filtration membrane, the supernatant was collected and recorded as crude enzyme fluid. The fluid was concentrated using an ultrafiltration centrifuge tube 10 times. A volume of 100 μL of the crude enzyme fluid was mixed with 900 μL of BR buffer solution (0.04 mol L^−1^, pH = 4), and the obtained sample was utilized in the protein adsorption process. The supernatant after adsorption and the recovered solution were collected and directed to a sulfate polyacrylamide gel electrophoresis (SDS-PAGE) assay to evaluate the isolation efficiency.

## 4. Conclusions

In summary, in the present study, water-soluble Co_4_PW was solidified into polydimethyldodecyl-4-vinylbenzylammonium chloride (PDDVAC) via a cation-exchange strategy to obtain an efficient sorbent, and the polyoxometalate (POM)-based polymeric ionic liquid composite was used in protein separation for the first time. The obtained Co_4_PW–PDDVAC composite was merited as having favorable biocompatibility and exhibited a high adsorption capacity towards PrK. Moreover, it is easy to collect and regenerate during the protein separation process. The selective adsorption of PrK on the Co_4_PW–PDDVAC composite was ascribed to the strong covalent coordination between the highly active Co^2+^ of polyoxometalate and the amino acid residue of PrK, as well as the synergistic multiple hydrogen bonds between the as-prepared composite and the target protein species. Compared with the commercial PrK isolation procedure, the Co_4_PW–PDDVAC-based solid-phased adsorption strategy has the advantages of a reduced isolation time and higher protein yield and activity, which might present a potential tool for large-scale PrK production. Moreover, composites with different morphologies and structures were obtained by regulating the molar ratios of polyoxometalate and polymeric ionic liquids. This not only provides a practical technique for the solidification of water-soluble polyoxometalate in meeting the demand to explore the structural diversity of polyoxometalate, but also offers a new perspective on designing and constructing multifunctional composites for bioanalytical applications. However, there are still some limitations in this study, such as the uneven pore size of the composite and the fact that the excellent amphoteric effect of the polymeric ionic liquid in the composite was not applied. Future work will focus on the preparation of bifunctional or multifunctional composites with a clear structure and uniform particle size.

## Figures and Tables

**Table 1:** Thermodynamic parameters for PrK adsorption by Co_4_PW–PDDVAC.

	T (K)	K_d_ (L g^−1^)	Δ*S^0^* (J mol^−1^)	Δ*H^0^* (KJ mol^−1^)	Δ*G^0^* (KJ mol^−1^)
PrK	288	6899.85	−98.48	−49.62	−21.26
	293	5005.98			−20.77
	303	2868.55			−19.78
	318	909.48			−18.30
	323	796.75			−17.81

**Table 2:** Isotherm parameters of PrK adsorption by Co_4_PW–PDDVAC composite.

Parameters	Linear	Nonlinear
Langmuir model		
b_L_ (L mg^−1^)	0.004794	0.002180
Q_m_ (mg g^−1^)	1428	2628
R^2^	0.9959	0.9951
Freundlich model		
n	1.295	1.457
K_F_ (mg g^−1^)	12.23	19.81
R^2^	0.9948	0.9899
Dubinin–Radushkevich model		
β (mol^2^ KJ^−2^)	3735	0.004795
Q_m_ (mg g^−1^)	1190	1503
R^2^	0.9128	0.9882
Temkin model		
K_T_ (L/mg)	0.06310	0.07886
B_T_ (KJ mol^−1^)	3.300	6.977
R^2^	0.9666	0.9666

**Table 3:** Adsorption kinetic parameters for PrK adsorption by Co_4_PW–PDDVAC composite.

Parameters	Linear	Nonlinear
Pseudo-first-order		
q_eq_ (mg g^−1^)	247.0	474.0
K_1_ (min^−1^)	0.4521	0.8240
R^2^	0.9944	0.9370
Pseudo-second-order		
q_eq_ (mg g^−1^)	512.0	526.0
K_2_ (g mg^−1^ min^−1^)	0.003151	0.002543
R^2^	0.9980	0.9720
Intraparticle		
K_3_ (mg g^−1^ min^−1/2^)	78.70	78.70
C (mg g^−1^)	265.0	265.0
R^2^	0.7761	0.7443
Elovich		
α	3402	4338
β	0.01200	0.01250
R^2^	0.9070	0.8800

**Table 4:** Comparisons of the performance of PrK isolation procedures.

	Commercial Procedure [51]	This Work
Isolation technique	Ammonium sulfate precipitate + Sephadex G-75 chromatography	Co_4_PW–PDDVAC-based solid-phase adsorption
Purification time	2–3 d	35 min (adsorption 5 min + elution 30 min)
Protein yield	2%	79.1%
Activity yield	28%	68.1%

## Data Availability

The data presented in this work are available in the article.

## Supplementary Materials

The following supporting information can be downloaded at: https://www.mdpi.com/article/10.3390/molecules28083307/s1. Figure S1: 1H-NMR spectra of ionic liquid DDVAC; Figure S2: 1H-NMR spectra of polymeric ionic liquid PDDVAC; Figure S3: Adsorption efficiency of proteinase K on PDDVAC/Co_4_PW molar ratio prepared from PDDVAC/Co_4_PW ratio of 1:1, 2:1, 5:1, 10:1, and 15:1; Figure S4: EDS mappings of element N (A), W (B), P (C), Co (D), C (E) and Co_4_PW-PDDVAC composite (F); Figure S5: (A) The amount of adsorbed PrK on Co_4_PW-PDDVAC composite under different temperatures, (B) the corresponding thermodynamic curve; Figure S6: Linear and nonlinear plots of pseudo-first-order equation kinetic model (A,B), pseudo-second-order equation kinetic model (C,D), intraparticle kinetic model (E,F), Elovich kinetic model (G,H) of PrK adsorption on Co_4_PW-PDDVAC composite; Table S1: Elemental analysis results of POM-PDDVAC composites.
