# Supplementary material for: Preparation of Polyoxometalate-Based Composite by Solidification of Highly Active Cobalt-Containing Polytungstate on Polymeric Ionic Liquid for the Efficient Isolation of Proteinase K
Source: Molecules. 2023 Apr 7;28(8):3307. doi: 10.3390/molecules28083307 (PMC10142915; doi:10.3390/molecules28083307)
Supplement: Supplementary file 1 [file molecules-28-03307-s001.zip › molecules-2318616-SI.pdf]

Supplementary Information

Preparation of polyoxometalate-based composite by solidification of high-active-cobalt-containing polytungstate on polymeric ionic liquid for the efficient isolation of Proteinase K

Jiaxuan Yang[1], Ning Chu[2], Xuwei Chen[1*]

[1] Department of Chemistry, College of Sciences, Northeastern University, Box 332, Shenyang 110819, China; y2627849213@163.com

[2] Bayuquan Customs District of the People's Republic of China, Yingkou 115007, China; n_chu2009@sina.com

* Correspondence: chenxuwei@mail.neu.edu.cn; Tel.: +86-24-83684533;

Fax: +86-24-83676698

Table of Contents

Figure S1. 1H-NMR spectra of ionic liquid DDVAC 2

Figure S2. 1H-NMR spectra of polymeric ionic liquid PDDVAC 2

Figure S3. Adsorption efficiency of proteinase K on PDDVAC/Co_4PW molar ratio prepared from PDDVAC/Co_4PW ratio of 1:1, 2:1, 5:1, 10:1, and 15:1. 3

Figure S4. EDS mappings of element N (A), W (B), P (C), Co (D), C (E) and Co_4PW-PDDVAC composite (F) 4

Figure S5. (A) The amount of adsorbed PrK on Co_4PW-PDDVAC composite under different temperature. (B) The corresponding thermodynamic curve........ 5

Figure S6. Linear and non-linear plots of pseudo first order equation kinetic model (A-B), pseudo second order equation kinetic model (C-D), intra particle kinetic model (E-F), Elovich kinetic model (G-H) of PrK adsorption on Co_4PW-PDDVAC composite.. 6

Table S1. Elemental analysis results of POM-PDDVAC composites 7

N,N-dimethyl-dodecyl-(4-vinylbenzyl) ammonium chloride (DDVAC): yellow wax solid. ^{1}H NMR (500 MHz, $CDCl_3$) δ 7.54 (d, *J*=8.0 Hz, 2H), 7.36 (d, *J*=8.0 Hz, 2H), 6.67 (dd, *J*=17.6, 10.9 Hz, 1H), 5.77 (d, *J*=17.6 Hz, 1H), 5.32 (d, *J*=10.9 Hz, 1H), 4.96 (s, 2H), 3.42 (dd, *J*=28.0, 19.7 Hz, 2H), 3.24-3.09 (m, 6H), 1.76 (s, 2H), 1.36-1.10 (m, 18H), 0.85 (t, *J*=7.0 Hz, 3H).

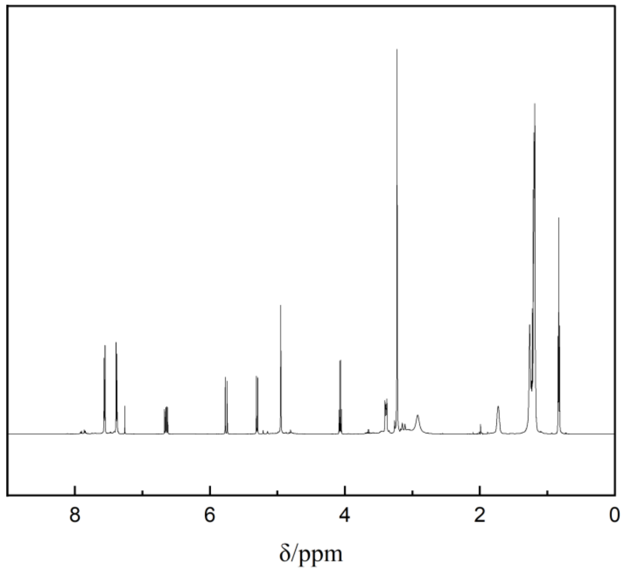

Figure S1. 1H-NMR spectra of ionic liquid DDVAC.

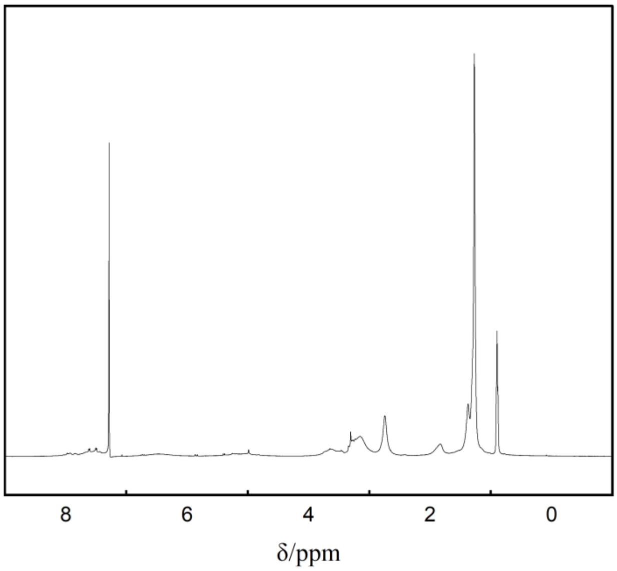

Figure S2. 1H-NMR spectra of polymeric ionic liquid PDDVAC.

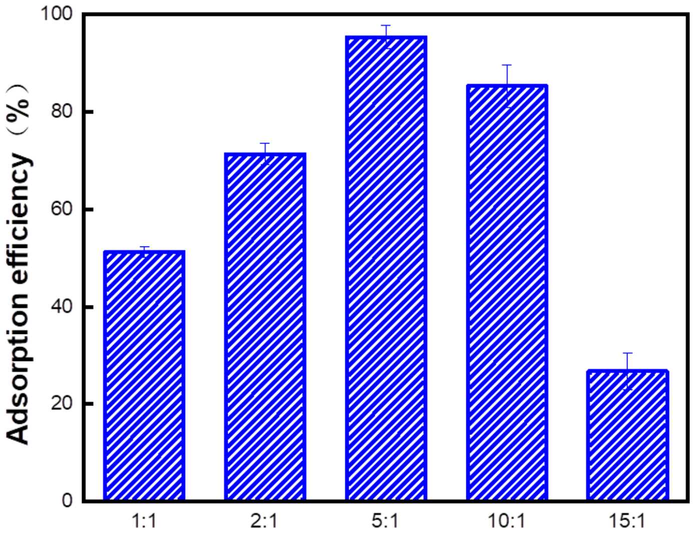

Figure S3. Adsorption efficiency of proteinase K on PDDVAC/Co_4PW molar ratio prepared from PDDVAC/Co_4PW ratio of 1:1, 2:1, 5:1, 10:1, and 15:1. Protein solution: 100 μg mL^{-1}, 1.0 mL; Co_4PW-PDDVAC composite: 0.2 mg; pH: 4.0.

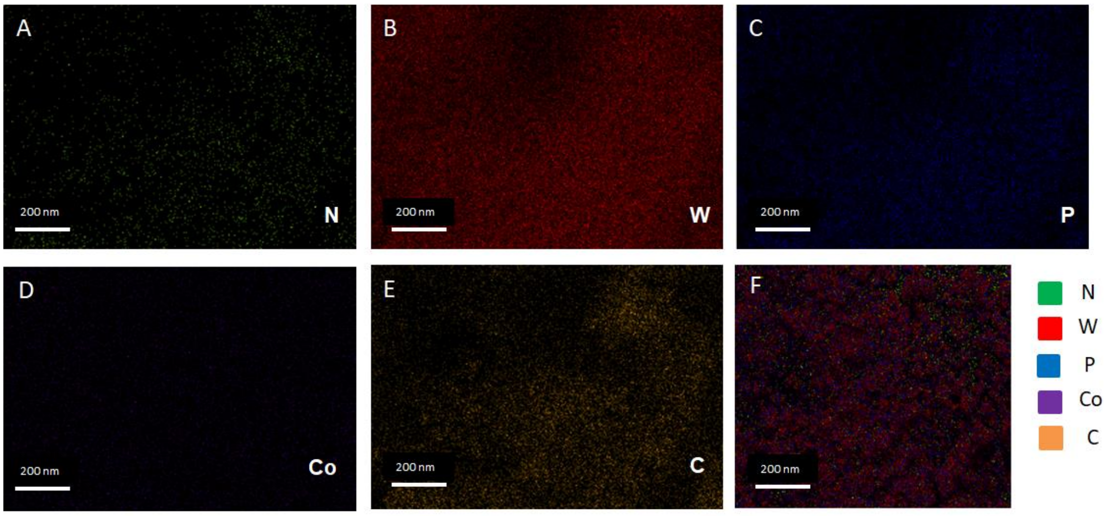

Figure S4. EDS mappings of element N (A), W (B), P (C), Co (D), C (E) and Co_4PW-PDDVAC composite (F).

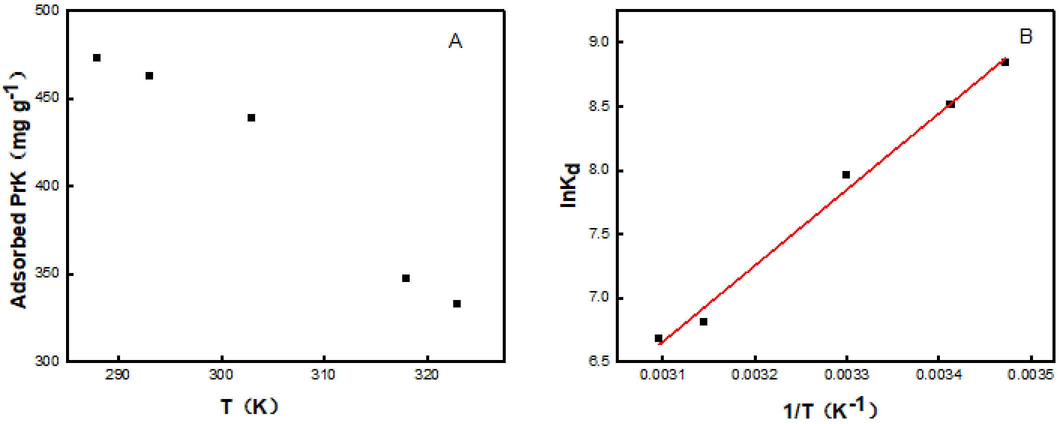

Figure S5. (A) The amount of adsorbed PrK on Co_4PW-PDDVAC composite under different temperature. (B) The corresponding thermodynamic curve.

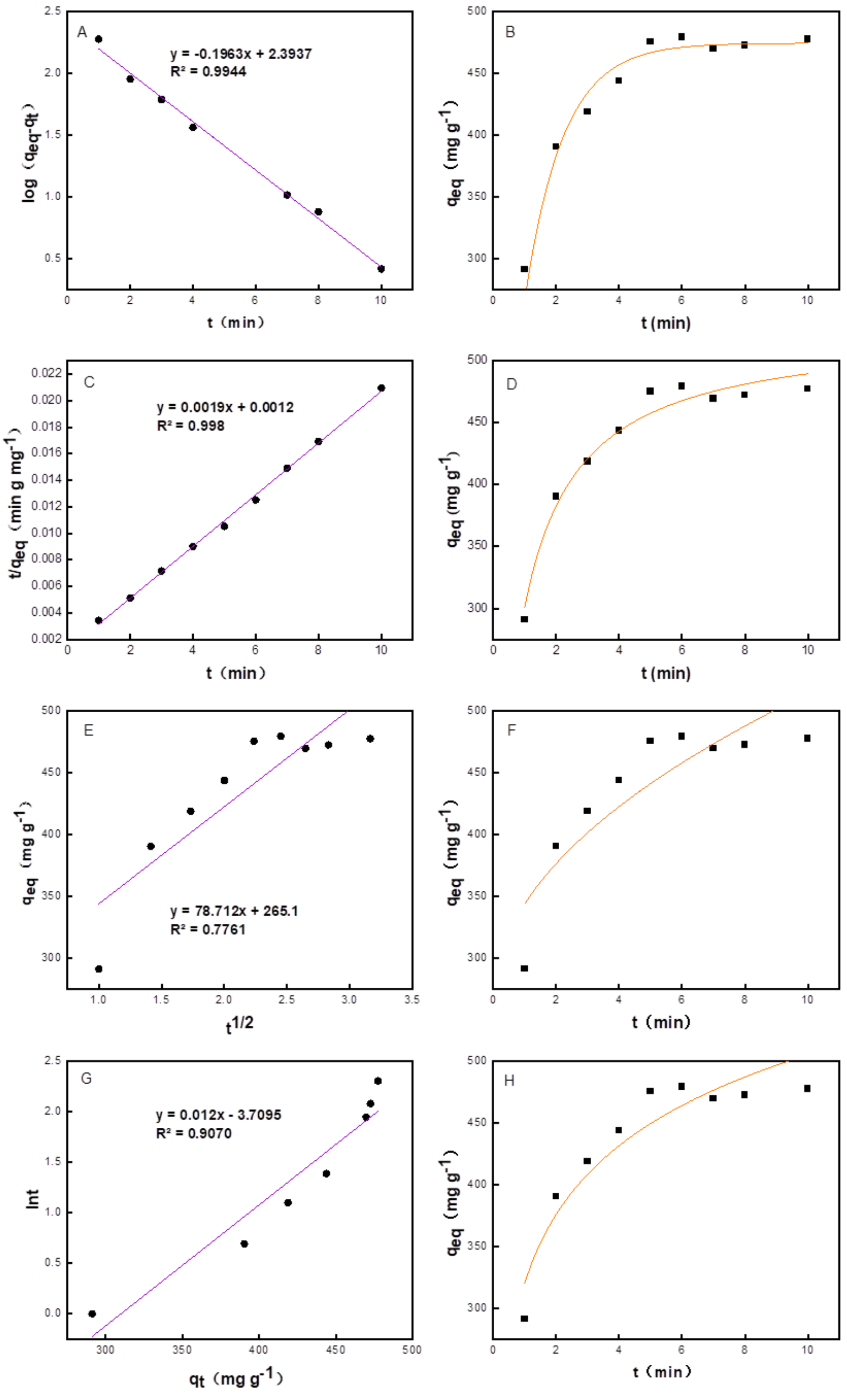

Figure S6. Linear and non-linear plots of pseudo first order equation kinetic model (A-B), pseudo second order equation kinetic model (C-D), intra particle kinetic model (E-F), Elovich kinetic model (G-H) of PrK adsorption on Co_4PW-PDDVAC composite.

Table S1. Elemental analysis results of POM-PDDVAC composites.

Element	CoPW-PDDVAC	Co_4PW-PDDVAC	Co_9PW-PDDVAC
Co/%	4.0	5.0	6.0
N/%	1.0	1.0	2.0
P/%	11.0	14.0	11.0
W/%	62.0	62.0	56.0
C/%	22.0	18.0	26.0
POM percentage/%	89.7	84.0	67.0
